# Primary Care Pathway for Childhood Asthma: Protocol for a Randomized Cluster-Controlled Trial

**DOI:** 10.2196/resprot.5261

**Published:** 2016-03-08

**Authors:** Andrew J Cave, Heather Sharpe, Mark Anselmo, A Dean Befus, Gillian Currie, Christina Davey, Neil Drummond, Jim Graham, Lee A Green, Jeremy Grimshaw, Karen Kam, Donna P Manca, Alberto Nettel-Aguirre, Melissa L Potestio, Brian H Rowe, Shannon D Scott, Tyler Williamson, David W Johnson

**Affiliations:** ^1^ Department of Family Medicine University of Alberta Edmonton, AB Canada; ^2^ Department of Paediatrics University of Calgary Calgary, AB Canada; ^3^ Alberta Children’s Hospital Research Institute University of Calgary Calgary, AB Canada; ^4^ Department of Medicine University of Alberta Edmonton, AB Canada; ^5^ Department of Community Health Sciences University of Calgary Calgary, AB Canada; ^6^ O’Brien Institute of Public Health University of Calgary Calgary, AB Canada; ^7^ Department of Family Medicine University of Calgary Calgary, AB Canada; ^8^ Alberta Health Services Edmonton, AB Canada; ^9^ Department of Medicine University of Ottawa Ottawa, ON Canada; ^10^ Ottawa Hospital Research Institute Ottawa, ON Canada; ^11^ Department of Emergency Medicine University of Alberta Edmonton, AB Canada; ^12^ School of Public Health University of Alberta Edmonton, AB Canada; ^13^ Faculty of Nursing University of Alberta Edmonton, AB Canada; ^14^ Department of Pharmacology and Therapeutics University of Calgary Calgary, AB Canada

**Keywords:** asthma, child, chronic disease management, primary care, clinical pathway, asthma education

## Abstract

**Background:**

Asthma is the most common chronic condition in children. For many, the disease is inadequately controlled, which can burden the lives of children and their families as well as the health care system. Improved use of the best available scientific evidence by primary care practitioners could reduce the need for hospital care and improve quality of life and asthma control, thereby reducing overall costs to society and families.

**Objective:**

The Primary Care Pathway for Childhood Asthma aims to improve the management of children with asthma by (1) providing primary care practitioners with an electronic guide (a clinical pathway) incorporated into the patient’s electronic medical record, and (2) providing train-the-trainer education to chronic disease management health professionals to promote the provision of asthma education in primary care.

**Methods:**

The research will utilize a pragmatic cluster-controlled design, quantitative and qualitative research methodologies, and economic evaluation to assess the implementation of a pathway and education intervention in primary care. The intervention will be analyzed for effectiveness, and if the results are positive, a strategy will be developed to implement delivery to all primary care practices in Alberta.

**Results:**

The research has been successfully funded and ethics approvals have been obtained. Practice recruitment began fall 2015, and we expect all study-related activities to be concluded by March 2018.

**Conclusions:**

The proposed pathway and education intervention has the potential to improve pediatric asthma management in Alberta. The intervention is anticipated to result in better quality of care for equal or lesser cost.

**ClinicalTrial:**

ClinicalTrials.gov NCT02481037; https://clinicaltrials.gov/ct2/show/NCT02481037 (Archived by WebCite at http://www.webcitation.org/6fPIQ02Ma).

## Introduction

### Background and Overview

Asthma is the most common chronic disease of childhood [1-5]. At least 15% of Canadian children are affected by this disease, and over 50% of these cases are poorly controlled [3-6]. Asthma is among the top 10 reasons for Albertan children to visit an emergency department and the top 5 reasons for urgent admissions to hospital [7,8]. As compared with healthy children, children with asthma miss three times as many school days [9,10] and have substantially reduced quality of life [11,12]. From a societal perspective, inadequate asthma control also presents a considerable economic burden due to the costs associated with hospital and emergency department admissions, medications, and caregiver productivity losses [13].

High-quality evidence exists regarding how to best manage childhood asthma in a primary care setting to optimize control and minimize acute disease exacerbations [14-16]. The best treatment varies depending on the child’s asthma disease phenotype, of which there are broadly two types. Although the terms used vary, we refer to these types as “persistent/seasonal disease” and “intermittent/viral triggered” [16-18]. Persistent/seasonal disease has continuous symptoms and is often triggered by allergens, exercise, cold air, and viral illnesses, whereas intermittent/viral-triggered disease is predominately provoked by viral upper respiratory tract infections and resolves completely between infections. For most children with persistent/seasonal disease, inhaled corticosteroids substantially reduce ongoing symptoms, bronchodilator use, asthma exacerbations, and health care utilization [14-17]. For children with intermittent/viral-triggered disease, montelukast (Singulair) has been shown to substantially reduce wheezing exacerbations, asthma symptoms, and health care utilization [19-21].

Although some studies have demonstrated methods to optimize asthma outcomes and minimize costs, this does not guarantee that patients actually receive the best care. It is estimated that across the spectrum of health care, 30-40% of patients do not receive treatments of proven effectiveness [22,23]. Studies from the United States have shown that primary care practitioners do not use preventive medications in up to 45% of children with asthma [24,25], and unpublished data (2008-2011) reported by *Alberta Health Services (AHS)* from Alberta pharmacies show that about 50% of children with asthma who are treated with rescue beta-agonists do not also receive preventative medications [26].

Clinical pathways are a common strategy for changing professional behavior and improving the quality of care delivery, reducing practice variation, and reducing costs of health care [27-30]. Although relatively few published studies have rigorously evaluated the impact of clinical pathways on clinical outcomes and costs [29], it is widely recognized that the rigor involved in pathway development and assessment of evidence obtained is important to improving outcomes. Accordingly, many health organizations such as AHS are using clinical pathways with this intent. In support of this approach, a clinical pathway focused on the emergency management of childhood asthma has been shown to increase the use of evidenced-based therapies [29,30]. However, creation of clinical pathways (or guidelines) is not enough; active implementation strategies are needed to achieve optimal improvements in outcomes [31-34]. Two strategies that are among the most consistently successful for changing practitioner behavior and improving patient outcomes are interactive educational sessions and reminder systems [31-34].

Even if children are prescribed appropriate medications, parents frequently do not receive adequate information and education to understand the rationale and importance of using preventive medications, which often results in poor adherence [35-37]. *Adherence* refers to a patient successfully taking medication. *Primary adherence* refers to the obtaining of medication prescribed, and *secondary adherence* refers to proper completion of the regime as intended by the prescriber. A World Health Organization [38] review indicated 30% adherence to medications by children and adolescents with asthma, which is among the lowest reported for any chronic disease. There is good evidence, however, that asthma education programs for children with severe exacerbations requiring emergency department and in-hospital care sufficiently improve adherence to reduce subsequent hospitalizations, emergency department visits, and urgent disease-related physician visits by 20-30% [39].

We propose to improve the prescription and use of evidenced-based preventative therapies for children with asthma, with the goal to significantly improve their disease control and quality of life, and reduce unnecessary emergency department visits and hospitalizations by 20% each. We will achieve this by (1) installing a primary care clinical pathway for managing childhood asthma into clinicians’ electronic medical record (EMR) to facilitate the use of best evidence by practitioners, and (2) training chronic disease management (CDM) health professionals to provide targeted and timely asthma education to parents and children with asthma. We will test this pathway and education project in a representative sample of 22 Alberta primary care practices, using a pragmatic cluster-controlled trial methodology. Specifically, we will determine if our proposed innovation, as compared with usual care,...

- ...increases *prescription* of evidenced-based preventative therapies;
- ...increases *dispensing* (primary adherence) of evidenced-based preventative therapies;
- ...improves children’s *asthma control*;
- ...improves *quality of life* for children and families; and
- ...decreases *health care utilization* (emergent primary care visits, emergency department visits, and hospitalizations) for asthma, and decreases overall societal (health system and family) costs.

### Foundational Activities

In Spring 2011, the *AHS* Respiratory Network—now formally known as the “Respiratory Health Strategic Clinical Network (RHSCN)”*—*adopted the childhood asthma pathway initiative, in collaboration with the *AHS* child health community, as one of its 3 top priorities and provided human and financial resources to move the pathway initiative forward. The initial project of the *RHSCN* and our project team was to develop, implement, and evaluate emergency department and in-patient pathways, for which we received the *Canadian Institutes of Health Research Partnerships for Health System Improvement* funding. Concurrently, with the start of the emergency department/in-patient pilot project and the ongoing implementation of the emergency department and in-patient pathways across the province, the *RHSCN* started developing the primary care pathway.

In Fall 2013, our team developed a collaboration with the *Health Quality Council of Alberta (HQCA)* who have agreed to extract, link, and analyze health administrative data (which they routinely receive and utilize) and other data provided to them from 2 Albertan community-based primary care research networks (Northern and Southern Alberta Primary Care Research Networks—*NAPCReN* [40] and *SAPCReN*, respectively 41). These networks are regional networks contributing to the *Canadian Primary Care Sentinel Surveillance Network* (*CPCSSN*) [42]. *CPCSSN* extracts clinical data quarterly from participating practitioners’ (*sentinels*) EMRs, cleans them, removes identifiers, and makes these data available for surveillance, epidemiology, and health services research. With the principal investigator (AC), *CPCSSN* is currently developing and validating a case definition and case finding algorithm for childhood asthma, which will be added in due course to its routine data extraction and processing. The networks contributing to *CPCSSN* in Alberta are currently able to process data from both the *Wolf* and *Med Access* EMR systems. These networks have agreed to assist with recruiting practices to participate in this project.

The project team has engaged extensively with frontline primary care practitioners, primary care leaders, and pediatric respirologists and allergists in developing this primary care pathway project over the last 3 years; each of these groups has heavily invested and is committed to the successful completion of our project. We have engaged frontline practitioners in the following ways: (1) being part of an interdisciplinary pathway development committee including primary care and subspecialty care practitioners with rural, regional, and urban representation; (2) conducting a small focus group (consultation) session about the content and format of the pathway with primary care practitioners at Alberta primary care conferences; (3) beta-testing of the EMR-based pathway in 4 primary care practices; and (4) surveying Alberta primary care practitioners for feedback about the pathway, its design, and practicality. The capacity to manage their asthma patients according to the best practice will be enhanced by the integration of the pathway into the EMRs and by the education given to the CDM health professionals. Participating clinics will identify an individual available for the practice who will take on the role of a CDM professional. The CDM professionals will receive a 3-hour training session/updating on asthma education with continued support from the trainer for the duration of the intervention. They will be provided with access to resources that will be available thereafter to ensure sustainability.

Currently, an asthma case definition is being developed, which is an algorithm that screens the *CPCSSN* data extracted from EMRs to accurately identify patients with asthma. Once developed, the case definition will be validated by comparing it with a gold standard established by 2 experienced physicians using 1000 pediatric *CPCSSN* records.

Network leadership will encourage their members’ participation in this initiative by introducing the study team to the medical and administrative leads of all their participating practices. Alongside *SAPCReN* and *NAPCReN* research staff, study team members will travel to eligible practice sites to inform them about the details of the project, answer any questions they might have, and then ask them to participate in this project.

### Key Stakeholders

In this project, the following 4 organizations will each play an essential role: AHS (*RHSCN)*, *HQCA*, and *NAPCReN/SAPCReN. RHSCN* and *HQCA* are committing substantial in-kind funding toward this project over the next 3 years. In addition, 3 other strategic clinical networks, namely, (1) *Maternal, Newborn, Child and Youth*, (2) *Emergency,* and (3) *Primary Care Chronic Disease Management*, share substantial common ground. A total of 3 primary care organizations, the 2 *AHS Primary and Community Care* portfolios and the *Provincial Primary Care Network*, will be “end users,” throughout the project, and will also provide critical advice as we plan our “implementation scale and spread” rollout. Participating primary care practices are also end users, providing valuable input into feasibility and sustainability. Finally, the patients of Alberta will be the final stakeholders, as we anticipate their health will be affected favorably.

## Methods

### Project Objectives

We propose a 3-year mixed-methods health services research project using a randomized cluster-controlled trial design (with primary care practices as the cluster; trial registration number NCT02481037) to achieve the following objectives:

- To use the Theoretical Domains Framework (TDF) to refine our implementation strategy;
- To implement the intervention (a childhood asthma primary care clinical pathway in EMRs and identifying and training CDM health professionals to provide asthma education) in the 11 practices in the intervention arm;
- To evaluate our project intervention using interviews, health administrative and EMR databases, and child and parent surveys of quality of life, the child’s asthma control, and the socioeconomic burden of the child’s disease on their family; and
- To develop a detailed “scale and spread” strategy if our intervention is shown to be successful (ie, yields either cost savings or improved child and parent quality of life) for all Alberta primary care practices who use a supported EMR.

### Intervention Development and Implementation Strategy

The development of our implementation strategy has followed the *Knowledge-to-Action Model* of Graham and colleagues [43]. This cyclic process starts with the identification of the problem—a knowledge-practice gap in the management of childhood asthma in a primary care setting—followed by synthesis of the evidence into a primary care pathway [18], assessment of potential barriers to its use, tailoring of known-effective implementation strategies to local circumstances, evaluation to determine whether the problem has improved and feedback of evaluation results to all end users (decision makers and participating practitioners).

The primary care pathway developed over 2 years by a subcommittee of the Asthma Working Group, RHSCN, was geographically representative of Alberta and included primary care practitioners (n=4), asthma educators (n=5), pharmacists (n=1), allergists and respiratory physicians (n=4), and asthma policy decision makers (n=2). This committee critically reviewed existing global and national guidelines [14,15] and recent randomized trials and systematic reviews to formulate the pathway. Drafts of the pathway were critically reviewed two times by primary care practitioners for ease of use and relevance to their practice, and revised each time.

The following sections provide a summary of the core components of the intervention to be provided to the intervention arm practices of the study:

#### Pathway Implementation Strategies

Based on previous extensive experience in implementing clinical pathways [44-46] and best evidence available [32,33,47-49], two change strategies were selected to implement the proposed project: (1) an interactive online learning module to teach primary care practitioners about the evidence for treatment strategies in the clinical pathway, and (2) user-written software installed within practitioners’ EMR that will serve as a reminder system to facilitate improved patient documentation and management. We will also use the results of a study being conducted using the TDF to identify necessary modifications to our strategy for primary care practices randomized to the intervention arm [50].

#### Development of EMR Embedded Pathway

The EMR prototype (developed in *Med Access* and currently being developed in *Wolf*) provides practitioners with dropdown menus to document key findings from medical history review and physical examination, classify patient’s disease phenotype to generate therapeutic recommendations, assess asthma control, generate a risk assessment, and print out asthma action plans and patient prescriptions.

#### Development of a Web-Based Interactive Learning Module

To ensure primary care practitioners understand the underlying rationale and evidence behind the development of the primary care pathway, practitioners randomized to the intervention arm will have easy access to a Web-based interactive learning module, which is designed to easily fit into a busy practitioner’s schedule. An equivalent module was developed by the research team and successfully used in a trial of an educational intervention in emergency room and in-patient management in Alberta [51]. Physicians and nurses will access the site with a password and will indicate completed sections before the intervention commences. A well-designed Web-based professional learning module has been shown in a previous trial to be as effective at enhancing learning and changing professional behavior as in-person interactive teaching sessions [52]. Parents will also be given access to patient-oriented Web modules to enhance the nurse-led teaching.

#### Train-the-Trainer for Patient Education

CDM health professionals working within the practices in the intervention arm will be provided 3 hours of in-person training on providing asthma education to children with asthma and their families. This training will be provided by the clinical coordinator, an experienced Certified Respiratory Educator (HS).

### Project Population

Practices will be recruited from those who are currently or are becoming a member of the *NAPCReN* or *SAPCReN.* All children (1-17 years of age at the time of enrollment) who meet the case definition of asthma (and are seen by a participating primary care practitioner within a consenting practice) will be included in the trial’s analysis unless they refuse consent [53]. The *CPCSSNs* use a “social contract” consent process, where practice populations are informed generally about research, information is freely available on specific projects, and people are included unless they object [53]. Because the project will collect data outside the *CPCSSN* protocol, parents of children with asthma in the participating practices will be sent a letter by their primary practitioner outlining implied consent and asking them to complete surveys about quality of life, asthma control, and the burden (impact) of asthma on their family.

### Sample Size Calculation and Analysis

Our *AHS* partners provided the number of pediatric asthma incidences billed by each practice in the province. We will use this information to select practices that have more than 70 active, eligible asthma patients. In addition, the Primary Care Asthma Working Group of clinicians agreed the minimal clinically important difference to be 20% (see Multimedia Appendix 1 for a detailed sample size calculation).

Patients included in the analysis of the primary and one of the secondary outcomes (proportion of children dispensed a preventive medication) will be categorized as either “successful” or “not successful” based on the logic outlined in Multimedia Appendix 2. Given the clustered nature of patients’ “success” within practices, a mixed effects logistic regression model will be performed where clustering is considered. Odds ratios for success between arms will be presented along with 95% CIs. The modeling will include assessment of potential confounders and independent risk factors. To analyze the other secondary outcomes (quality of life and asthma control and emergency department visits/hospitalizations), we will use a linear mixed effects regression model, and include fixed effects for the baseline/follow-up periods, the pathway/control arms, and stratifications/interaction, and random effects to account for clustering in practices.

### Randomization of Practices Design Issues and Potential Bias

All practices consenting to participate in this trial will be randomized, stratified for urban (Edmonton/Calgary) versus nonurban and academic (trainees routinely evaluate patients) versus nonacademic practices. A statistician not aware of our project question will allocate practices using a standard randomization software. We have chosen practices as the unit of randomization because most individual practitioners within a shared practice use the same EMR and we wish to prevent contamination. Because we are testing the effectiveness of embedding the pathway into clinicians’ EMR and train-the-trainer CDM professional education, consenting practices randomized to usual care will not have access to either intervention, and therefore the risk of significant contamination is small. In addition, the design provides an opportunity to test for contamination by comparing the difference between the pre- and post-periods for the 2 groups (ie, intervention and control).

Practices will be randomized to either:

- Routine care versus intervention
- Embedding a primary care clinical pathway for managing childhood asthma into clinicians’ EMR to facilitate practitioners utilizing best evidence, and training these practices’ CDM professionals to provide asthma education to children with asthma and their parents.

### Quantitative Data

Data will come from 3 sources, namely, health administrative sets, data extracted from participating practices’ EMR, and surveys of asthma control, quality of life, and socioeconomic factors. Health administrative datasets will include Alberta Patient Registry, emergency department visits (*National Ambulatory Care Reporting System*), hospitalization abstracts (*Discharge Abstract Database*), practitioners visits (*Alberta physician/NP claims*), and pharmacy dispensing (*Pharmaceutical Information Network*); these datasets are considered to be of good quality with accurate personal identifiers on more than 95% of records. As previously noted, EMR data using a validated case definition will be extracted for all children with asthma meeting the inclusion/exclusion criteria. This will provide individual categorical and continuous patient-level data such as patient demographics, encounter history, medication prescriptions, and comorbid disease. Also as previously noted, the *HQCA* will play a pivotal role in our trial as they will (1) receive the aforementioned datasets from *AHS*, Alberta Health, and *NAPCReN/SAPCReN*; (2) extract and link the necessary data using a separate file from the practitioners, which will contain patients’ *CPCSSN* identifier linked to their Alberta Health Care Number; (3) perform the planned analysis; and (4) provide the aggregated results to our research team.

Letters from participating practitioners will be sent two times to families of children with asthma before and 11 months after the study intervention starts. The letter will inform parents about the purpose of the survey (though the broad purpose of the project will not be revealed to maintain masking) and will ask the primary parent or caretaker to go online to review the information sheet and complete a socioeconomic survey and the *Paediatric Asthma Caregiver’s Quality of Life Questionnaire (PACQLQ)* for children with asthma, or to complete written copies of the questionnaires and return them by self-addressed stamped envelopes. A previous study [54] has validated the *PACQLQ* for use by parents of children less than 7 years of age. Children 7 years and older will be asked to also complete the *Standardised Paediatric Asthma Quality of Life Questionnaire* and the *Asthma Control Questionnaire* [55]. The socioeconomic survey includes questions such as day care or school days missed, parent work days missed, and asthma medications purchased in the last 30 days, to allow calculation of the economic impact of a child’s asthma on their family.

### Qualitative Data

We will purposefully select 6 practices randomized to the pathway and education intervention based on size of the practice and heterogeneity of professionals in the practice [56], and evaluate them using multidisciplinary focus groups after the intervention. The interview protocols will be developed following the implementation of the intervention to incorporate key learning from the implementation into the question development. The focus groups will include practitioners and other professionals, for a total of at least six clinicians, and will be recorded by a court reporter [57]. The content of the interview questions will be guided by the Knowledge to Action Model [58] and will target areas such as attributes of the pathway, adopters, and implementation setting. Clinicians will have the opportunity to provide feedback regarding potential challenges with the pathway and offer suggestions for improvements.

To monitor the progress and permit follow-up of ideas that emerge from the focus group, data collection and analysis will follow an iterative and concurrent process. The post-implementation focus group data will be analyzed in the following three phases: coding, categorizing, and developing themes [59]. First, data for each practice will be coded to facilitate analysis. The code word(s) will reflect the essence of the data leading to ease of recognition as the number of code words increases. Codes will be operationally defined so that they can be consistently applied throughout the data. Second, codes will be placed into broad categories that correspond to the major unit of analysis. As categories emerge, their theoretical properties will be defined. Third, comparisons between categories will be carried out to locate similarities and differences among them. Data analysis will be carried out using NVivo 10.

### Economic Analysis

The potential for provincial-wide savings is achievable because of the high-quality evidence (numerous randomized controlled trials, systematic reviews, and international consensus guidelines) demonstrating that appropriately tailored anti-inflammatory medications reduce emergency department visits and hospitalizations and that asthma education training targeted to focus on families with children with more severe disease also yields a 20% reduction in subsequent hospitalizations, a 30% reduction in emergency department visits, and a 30% reduction in nonscheduled urgent physician visits for disease exacerbation [39]. Further validating the ability of preventative therapy for asthma to reduce health care utilizations and costs, health services studies in children and adults in both British Columbia and Sweden utilizing administrative data have shown that higher utilization of inhaled corticosteroids is correlated with lower rates of hospitalization and lower overall health care costs [60,61].

The primary analysis will be from the health care system perspective. Total health system costs per child with asthma in the 2 trial arms before and after the intervention will be determined. Health system costs will be disaggregated into the following three parts: (1) those stemming from pathway implementation and train-the-trainer education sessions, 2) those from asthma education provided by CDM professionals, and (3) those from hospitalizations and emergency department and practitioner visits for asthma. Overall health system costs will be considered decreased by the project intervention if the cost decrease from reduced emergent physician visits, emergency department visits, and hospitalization outweighs the cost increase from pathway implementation and asthma education. The impact on costs will be considered alongside the impact on outcomes including quality of life. A secondary analysis will also include consideration of family costs that consist of parent loss of work, travel, or other out-of-pocket costs due the child’s disease.

Costs stemming from health care utilization (drugs, hospitalizations, emergency department, and practitioner visits) will be derived from health administrative data. Actual costs are available from *AHS* for in-patient stays at Calgary and Edmonton Zone hospitals, and costs for other Alberta hospitals can be approximated using Institute of Health Economics (IHE) provincial estimates [62]. Emergency department visit costs will be determined using the IHE standard provincial estimates [62]. Surveys of staff and parents of children with asthma from each of the participating practices will provide cost estimates by collecting the following data: time spent on train-the-trainer sessions, staff time to educate families, and family quality of life measures, as well as out-of-pocket expenditures on their child’s disease.

### Ethics Approval

The protocol obtained approval from the Health Research Ethics Board at the University of Alberta (Edmonton, AB) on August 7, 2015, and the Conjoint Health Research Ethics Board at the University of Calgary (Calgary, AB) on September 23, 2015.

## Results

Practice recruitment began September 2015, and all study-related activities are expected to conclude March 2018 (see Multimedia Appendix 3 for a timeline of major objectives) with the following outcomes:

### Primary Outcome

The proportion of symptomatic children with asthma in the baseline and follow-up periods (separate calculations) who are appropriately treated with a preventer will be the primary outcome. The denominator will be the number of children who meet the case definition of asthma *and* receive at least one prescription for an inhaled short-acting beta-agonist (eg, salbutamol) during the applicable period. The numerator will be, of these children, the number of those who receive a prescription for inhaled corticosteroid, montelukast, a combination of inhaled long-acting beta-agonist and corticosteroid (ie, agonist + corticosteroid), or some combination of these 3 drugs in the same period.

### Secondary Outcomes

#### Dispensed Preventative Therapies (Primary Adherence)

The proportion of applicable children in baseline and follow-up periods who are appropriately *dispensed* a preventer will highlight if there is a significant gap between prescriptions given and filled. The denominator will be the same as for the primary outcome but the numerator will be the number of these children who are *dispensed* 1 or more preventer medications from newly available PIN data.

#### Emergency Department Visits and Hospitalizations for Asthma

The number of asthma emergency department visits or hospitalizations for asthma (ICD10 J45 or J46) per child who meets the case definition of asthma during each period will be a measure of health care use.

#### Electronic Medical Record Data

The *CPCSSN* and research team will develop and validate a case definition and case finding algorithm for identifying children with asthma in the *CPCSSN* practices. The *CPCSSN* EMR data will provide, for all eligible children, individual categorical and continuous patient-level data such as patient demographics, ICD9 codes, and medication prescriptions. For children evaluated in practices randomized to the pathway group, data will also include asthma phenotype and provision of asthma action plans.

## Discussion

The management of pediatric asthma in Alberta is variable in quality with only half of the affected children having control of their asthma. This has adverse effects on morbidity and costs of health care. We aim to improve the quality of care in primary care by a two-pronged approach: the use of a management pathway in the practitioner’s EMR and the education of parents in the importance of preventive therapies.

At the end of the 3-year project, the rigorous pragmatic cluster-controlled design will provide a thorough and comprehensive understanding of whether this project improves patient care, how effective the interventions are, and whether the interventions add value or are cost saving. If the results are positive, we will collaborate with the *AHS RHSCN,* the *Provincial Primary and Community Care* portfolios, and the *Provincial Primary Care Network* to deliver this pathway and educational initiative to all Alberta primary care practices and their patients.

## Appendix

Multimedia Appendix 1 Sample size sensitivity analysis.

Multimedia Appendix 2 Logic model for determining â€˜overall successâ€™ (appropriate treatment of symptomatic asthmatic child with preventer medication) for primary outcome.

Multimedia Appendix 3 Timeline of major objectives and associated milestones.

Multimedia Appendix 4 Peer-review from funding agency.

## Abbreviations

AHS: Alberta Health Services
CDM: chronic disease management
CPCSSN: Canadian Primary Care Sentinel Surveillance Network
EMR: electronic medical record
HQCA: Health Quality Council of Alberta
IHE: Institute of Health Economics
NAPCReN: Northern Alberta Primary Care Research Networks
PACQLQ: Paediatric Asthma Caregiver’s Quality of Life Questionnaire
RHSCN: Respiratory Health Strategic Clinical Network
SAPCReN: Southern Alberta Primary Care Research Networks
TDF: Theoretical Domains Framework

## References

[1] Akinbami LJ, Moorman JE, Bailey C, Zahran HS, King M, Johnson CA, Liu X (2012). Trends in asthma prevalence, health care use, and mortality in the United States, 2001-2010. NCHS Data Brief.

[2] Torpy JM, Campbell A, Glass RM (2010). JAMA patient page. Chronic diseases of children. JAMA.

[3] Gershon AS, Guan J, Wang C, To T (2010). Trends in asthma prevalence and incidence in Ontario, Canada, 1996-2005: a population study. Am J Epidemiol.

[4] Hessel PA, Klaver J, Michaelchuk D, McGhan S, Carson MM, Melvin D (2001). The epidemiology of childhood asthma in Red Deer and Medicine Hat, Alberta. Can Respir J.

[5] Garner R, Kohen D (2008). Changes in the prevalence of asthma among Canadian children. Health Rep.

[6] McGhan SL, MacDonald C, James DE, Naidu P, Wong E, Sharpe H, Hessel PA, Befus AD (2006). Factors associated with poor asthma control in children aged five to 13 years. Can Respir J.

[7] Moser D (2012). Alberta Health Services Emergency Department Visits and Hospitalizations for Children 0-18 Years.

[8] To T, Dell S, Dick P, Cicutto L (2008). The burden of illness experienced by young children associated with asthma: a population-based cohort study. J Asthma.

[9] Fowler MG, Davenport MG, Garg R (1992). School functioning of US children with asthma. Pediatrics.

[10] Taylor WR, Newacheck PW (1992). Impact of childhood asthma on health. Pediatrics.

[11] Merikallio VJ, Mustalahti K, Remes ST, Valovirta EJ, Kaila M (2005). Comparison of quality of life between asthmatic and healthy school children. Pediatr Allergy Immunol.

[12] Kojima N, Ohya Y, Futamura M, Akashi M, Odajima H, Adachi Y, Kobayashi F, Akasawa A (2009). Exercise-induced asthma is associated with impaired quality of life among children with asthma in Japan. Allergol Int.

[13] Ungar WJ, Coyte PC, Pharmacy Medication Monitoring Program Advisory Board (2001). Prospective study of the patient-level cost of asthma care in children. Pediatr Pulmonol.

[14] Global Initiative for Asthma (GINA) (2015). From the Global Strategy for Asthma Management and Prevention.

[15] Lougheed MD, Lemiere C, Ducharme FM, Licskai C, Dell SD, Rowe BH, Fitzgerald M, Leigh R, Watson W, Boulet LP, Canadian Thoracic Society Asthma Clinical Assembly (2012). Canadian Thoracic Society 2012 guideline update: diagnosis and management of asthma in preschoolers, children and adults. Can Respir J.

[16] Kovesi T, Schuh S, Spier S, Bérubé D, Carr S, Watson W, McIvor RA (2010). Achieving control of asthma in preschoolers. CMAJ.

[17] Baraldi E, Bisgaard H, Boner AL, Castro-Rodriguez JA, Custovic A, de Blic J, Eber E, Everard ML, Frey U, Gappa M, Garcia-Marcos L, Grigg J, Lenney W, Le Souëf P, McKenzie S, Midulla F, Paton JY, Piacentini G, Pohunek P, Rossi GA, Seddon P, Silverman M, Sly PD, Stick S, Valiulis A, Wildhaber JH, Wennergren G, Wilson N, Zivkovic Z, Bush A, Brand PLP, de Jongste JC, Merkus PJFM, van Aalderen WMC (2008). Definition, assessment and treatment of wheezing disorders in preschool children: an evidence-based approach. Eur Respir J.

[18] Alberta Childhood Asthma Pathway Working Group (2014). Alberta Childhood Asthma Pathway for Primary Care.

[19] Johnston NW, Mandhane PJ, Dai J, Duncan JM, Greene JM, Lambert K, Sears MR (2007). Attenuation of the September epidemic of asthma exacerbations in children: a randomized, controlled trial of montelukast added to usual therapy. Pediatrics.

[20] Robertson CF, Price D, Henry R, Mellis C, Glasgow N, Fitzgerald D, Lee AJ, Turner J, Sant M (2007). Short-course montelukast for intermittent asthma in children: a randomized controlled trial. Am J Respir Crit Care Med.

[21] Bisgaard H, Zielen S, Garcia-Garcia ML, Johnston SL, Gilles L, Menten J, Tozzi CA, Polos P (2005). Montelukast reduces asthma exacerbations in 2- to 5-year-old children with intermittent asthma. Am J Respir Crit Care Med.

[22] Schuster MA, McGlynn EA, Brook RH (1998). How good is the quality of health care in the United States?. Milbank Q.

[23] Grol R (2001). Successes and failures in the implementation of evidence-based guidelines for clinical practice. Med Care.

[24] Diette GB, Skinner EA, Markson LE, Algatt-Bergstrom P, Nguyen TT, Clark RD, Wu AW (2001). Consistency of care with national guidelines for children with asthma in managed care. J Pediatr.

[25] Diette GB, Skinner EA, Nguyen TT, Markson L, Clark BD, Wu AW (2001). Comparison of quality of care by specialist and generalist physicians as usual source of asthma care for children. Pediatrics.

[26] Alberta Health Services (2013). Report on the Use of Asthma Medications in Albertan Children and Adults, 2008-2011.

[27] Schrijvers G, van Hoorn A, Huiskes N (2012). The care pathway: concepts and theories: an introduction. Int J Integr Care.

[28] Campbell H, Hotchkiss R, Bradshaw N, Porteous M (1998). Integrated care pathways. BMJ.

[29] Rotter T, Kinsman L, James E, Machotta A, Gothe H, Willis J, Snow P, Kugler J (2010). Clinical pathways: effects on professional practice, patient outcomes, length of stay and hospital costs. Cochrane Database Syst Rev.

[30] Cunningham S, Logan C, Lockerbie L, McMurray A, Prescott RJ, Dunn MJG (2008). Effect of an integrated care pathway on acute asthma/wheeze in children attending hospital: cluster randomized trial. J Pediatr.

[31] Grimshaw JM, Shirran L, Thomas R, Mowatt G, Fraser C, Bero L, Grilli R, Harvey E, Oxman A, O'Brien MA (2001). Changing provider behavior: an overview of systematic reviews of interventions. Med Care.

[32] Grimshaw JM, Thomas RE, MacLennan G, Fraser C, Ramsay CR, Vale L, Whitty P, Eccles MP, Matowe L, Shirran L, Wensing M, Dijkstra R, Donaldson C (2004). Effectiveness and efficiency of guideline dissemination and implementation strategies. Health Technol Assess.

[33] Bero LA, Grilli R, Grimshaw JM, Harvey E, Oxman AD, Thomson MA (1998). Closing the gap between research and practice: an overview of systematic reviews of interventions to promote the implementation of research findings. The Cochrane Effective Practice and Organization of Care Review Group. BMJ.

[34] Grimshaw J, Eccles M, Russell I (1995). Developing clinically valid practice guidelines. J Eval Clin Pract.

[35] Haynes RB, Ackloo E, Sahota N, McDonald HP, Yao X (2008). Interventions for enhancing medication adherence. Cochrane Database Syst Rev.

[36] Janson SL, McGrath KW, Covington JK, Cheng SC, Boushey HA (2009). Individualized asthma self-management improves medication adherence and markers of asthma control. J Allergy Clin Immunol.

[37] Drotar D, Bonner MS (2009). Influences on adherence to pediatric asthma treatment: a review of correlates and predictors. J Dev Behav Pediatr.

[38] Sabate E (2003). Adherence to Long-Term Therapies: Evidence for Action.

[39] Boyd M, Lasserson TJ, McKean MC, Gibson PG, Ducharme FM, Haby M (2009). Interventions for educating children who are at risk of asthma-related emergency department attendance. Cochrane Database Syst Rev.

[40] Northern Alberta Primary Care Research Network (NAPCReN) NAPCReN: About [Home Page].

[41] Southern Alberta Primary Care Research Network (SAPCReN) SAPCReN: About [Home Page].

[42] Canadian Primary Care Sentinel Surveillance Network (CPCSSN) CPCSSN: About [Home Page].

[43] Graham ID, Logan J, Harrison MB, Straus SE, Tetroe J, Caswell W, Robinson N (2006). Lost in knowledge translation: time for a map?. J Contin Educ Health Prof.

[44] Jabbour M, Curran J, Scott SD, Guttman A, Rotter T, Ducharme FM, Lougheed MD, McNaughton-Filion ML, Newton A, Shafir M, Paprica A, Klassen T, Taljaard M, Grimshaw J, Johnson DW (2013). Best strategies to implement clinical pathways in an emergency department setting: study protocol for a cluster randomized controlled trial. Implement Sci.

[45] Johnson DW, Craig W, Brant R, Mitton C, Svenson L, Klassen TP (2006). A cluster randomized controlled trial comparing three methods of disseminating practice guidelines for children with croup [ISRCTN73394937]. Implement Sci.

[46] Stoloff SW, Srebro SH, Edwards LD, Johnson MC, Rickard KA (2001). Improved asthma control after changing from low-to-medium doses of other inhaled corticosteroids to low-dose fluticasone propionate. MedGenMed.

[47] Cook DA, Levinson AJ, Garside S, Dupras DM, Erwin PJ, Montori VM (2008). Internet-based learning in the health professions: a meta-analysis. JAMA.

[48] Chaudhry B, Wang J, Wu S, Maglione M, Mojica W, Roth E, Morton SC, Shekelle PG (2006). Systematic review: impact of health information technology on quality, efficiency, and costs of medical care. Ann Intern Med.

[49] Garg AX, McDonald H, Rosas-Arellano MP, Devereaux PJ, Beyene J, Sam J, Haynes RB, Adhikari NKJ (2005). Effects of computerized clinical decision support systems on practitioner performance and patient outcomes: a systematic review. JAMA.

[50] Cane J, O'Connor D, Michie S (2012). Validation of the theoretical domains framework for use in behaviour change and implementation research. Implement Sci.

[51] Alberta Acute Childhood Pathways Teaching: About [Home Page].

[52] Fordis M, King JE, Ballantyne CM, Jones PH, Schneider KH, Spann SJ, Greenberg SB, Greisinger AJ (2005). Comparison of the instructional efficacy of Internet-based CME with live interactive CME workshops: a randomized controlled trial. JAMA.

[53] Singleton P, Wadsworth M (2006). Consent for the use of personal medical data in research. BMJ.

[54] Osman LM, Baxter-Jones AD, Helms PJ (2001). Parents' quality of life and respiratory symptoms in young children with mild wheeze. EASE Study Group. Eur Respir J.

[55] Juniper EF, Gruffydd-Jones K, Ward S, Svensson K (2010). Asthma Control Questionnaire in children: validation, measurement properties, interpretation. Eur Respir J.

[56] Scott SD, Plotnikoff RC, Karunamuni N, Bize R, Rodgers W (2008). Factors influencing the adoption of an innovation: an examination of the uptake of the Canadian Heart Health Kit (HHK). Implement Sci.

[57] Scott SD, Sharpe H, O'Leary K, Dehaeck U, Hindmarsh K, Moore JG, Osmond MH (2009). Court reporters: a viable solution for the challenges of focus group data collection?. Qual Health Res.

[58] Graham ID, Logan J (2004). Innovations in knowledge transfer and continuity of care. Can J Nurs Res.

[59] Morse JM, Field PA (1995). Qualitative Research for Health Professionals. Second edition.

[60] Gerdtham UG, Hertzman P, Jönsson B, Boman G (1996). Impact of inhaled corticosteroids on acute asthma hospitalization in Sweden 1978 to 1991. Med Care.

[61] Bedouch P, Sadatsafavi M, Marra CA, FitzGerald JM, Lynd LD (2012). Trends in asthma-related direct medical costs from 2002 to 2007 in British Columbia, Canada: a population based-cohort study. PLoS One.

[62] Jacobs P, Bachynsky J (1997). An Alberta Standard Cost List for Health Economics Evaluations.

