# Supplementary material for: Primary Care Pathway for Childhood Asthma: Protocol for a Randomized Cluster-Controlled Trial
Source: JMIR Res Protoc. 2016 Mar 8;5(1):e37. doi: 10.2196/resprot.5261 (PMC4804104; doi:10.2196/resprot.5261)
Supplement: Multimedia Appendix 1 [file resprot_v5i1e37_app1.pdf]

The minimum projected sample size represents the number of primary practices required if:

1) the average number of children evaluated per year with asthma in a practice is the same;
2) the intercluster correlation coefficient is smaller than anticipated;
3) the baseline proportion of children treated with preventer medications is significantly worse than expected; and
4) we assume the minimum clinically important difference is bigger than 15%.

The maximum projected sample size represents the number of primary practices required if:

1) average number of children evaluated per year with asthma in a practice is the same;
2) intercluster correlation coefficient is larger than anticipated;
3) the baseline proportion of children treated with preventer is significantly better than expected; and
4) we assume the minimum clinically important difference is smaller than 15%.

	**Minimum**	**Maximum**
**Cluster Size**	**70**	**70**
**ICC**	**0.02**	**0.03**
**Baseline % treated**	**0.10**	**.50**
**Minimal Clinically Important Difference**	**0.20**	**.10**
**Number of Clusters Required**	**14**	**44**

Sample size calculation: To calculate our sample size using our primary outcome - proportion of symptomatic asthmatic children in the baseline and follow-up periods that are appropriately treated with a preventer - we used data from all Alberta primary care practices that use *Med Access* as their EMR. The median number of children (< 18 yrs) with asthma evaluated each year in these practices is 70, the estimated proportion of 'success' is 30%, and the estimated intraclass correlation coefficient (ICC) is 0.025 (which is consistent with ICC values reported for primary care research) [1]. To estimate the minimal clinically significant change for this trial, we conducted a survey of practitioners which determined that a 15% absolute change in preventer treatment was clinically important. Using the above values (cluster size = 70, ICC = 0.025, proportion of 'success' in control arm = 30%, minimal clinically important improvement = + 15%, that is, proportion of success in intervention arm=45%) and the approach of Hayes and Bennett [2], we determined 11 clusters per arm (or 22 practices) would provide us with more than 90% power, setting an alpha of 0.05. This power suggests our sample size is robust.

Design Justification: Based on recommendations by Donner, we will not use a matched pair design because of lack of baseline information on the primary outcome, rate of preventive therapy in practices [3-4]. Although our project intervention has 2 components (pathway and CDM professional training), we have chosen not to use a factorial study design because these components will be highly interwoven, and, we anticipate, synergistic. A review of factorial studies highlights that if synergy between interventions occurs [5], factorial design can result in inadequate power to detect differences between

interventions.

References for Sample Size Calculation

1. Adams G, Gulliford MC, Ukoumunne OC, Eldridge S, Chinn S, Campbell MJ. Patterns of intra-cluster correlation from primary care research to inform study design and analysis. *J Clin Epidemiol*. 2004;57(8):785–794. PMID: 15485730.
2. Hayes RJ, Bennett S. Simple sample size calculation for cluster-randomized trials. *Int J Epidemiol*. 1999;28(2):319–326. PMID: 10342698.
3. Klar N, Donner A. Current and future challenges in the design and analysis of cluster randomization trials. *Stat Med*. 2001;20(24):3729–3740. PMID: 11782029.
4. Donner A. The role of cluster randomization trials in health research. URL: http://www.newton.ac.uk/programmes/DAE/seminars/2011081514001.pdf. (Archived by WebCite® at http://www.webcitation.org/6eVpa0bOU)
5. Byth K, Gebski V. Factorial designs: a graphical aid for choosing study designs accounting for interaction. *Clin Trials*. 2004;1(3):315–325. PMID: 16279257.
