# Supplementary material for: Primary Care Pathway for Childhood Asthma: Protocol for a Randomized Cluster-Controlled Trial
Source: JMIR Res Protoc. 2016 Mar 8;5(1):e37. doi: 10.2196/resprot.5261 (PMC4804104; doi:10.2196/resprot.5261)
Supplement: Multimedia Appendix 2 [file resprot_v5i1e37_app2.pdf]

We estimate that approximately 60% of patients will be evaluated by their practitioner in both the baseline and follow-up time periods. The remaining patients will be analyzed in only the baseline or follow-up period but not the other. This asymmetry requires a consistent method for classifying ‘baseline’ effects for both pathway and control arms. We will use the method outlined below:

	Patient Evaluated In This Time Period		Interpretation S vs. NS
	Baseline	Follow-up	
Both	Y	Y	S
	N	Y	S
	Y	N	NS
Baseline Only	Y	-	S
	N	-	NS
Follow-up Only	-	Y	S
	-	N	NS

Y or N = Patient evaluated during this time period

- = Patient **not** evaluated during this time period

Y = Yes, appropriately treated with preventer **in this time period**

N = No, **not** appropriately treated with preventer **in this time period**

S = Success, appropriately treated with preventer **overall**

NS = Non-Success, **not** appropriately treated with preventer **overall**
