# Supplementary material for: Primary Care Pathway for Childhood Asthma: Protocol for a Randomized Cluster-Controlled Trial
Source: JMIR Res Protoc. 2016 Mar 8;5(1):e37. doi: 10.2196/resprot.5261 (PMC4804104; doi:10.2196/resprot.5261)
Supplement: Multimedia Appendix 4 [file resprot_v5i1e37_app4.pdf]

Alberta Innovates Health Solutions

201408 Partnership for Research & Innovation In the Health System Competition

Lead: Cave, Andrew J.

Co-Lead(s): Johnson, David W.
Grimshaw, Jeremy

Record #: 201400389

Project Title: Primary Care Pathway for Childhood Asthma

Your application was reviewed by the PRIHS Review Committee and ranked within the fundable pool. Please find additional comments/feedback from the process below.

Committee Meeting Comments:

- Thoughtful consideration to the medication adherence measure is encouraged
- Good qualitative component; however, this will not inform the causal chain of patient adherence or address barriers to change at the patient level (i.e. medication cost)
- The team is encouraged to examine additional outcome measures to strengthen the study
- Well established group with a good track record and a cohesive approach
- Potential for significant cost savings to the system
